# Electrospun PMVEMA Nanofibers Developed as a Fast-Release Platform for Antineoplastic Drugs Tested in Glioblastoma Primary Cultures

**DOI:** 10.3390/pharmaceutics17091172

**Published:** 2025-09-08

**Authors:** Pedro Valentín Badía-Hernández, Joan Moll Carrió, María Fuentes-Baile, María Losada-Echeberría, Rocío Díaz-Puertas, Amalia Mira, Miguel Saceda, Pilar García-Morales, Ricardo Mallavia

**Affiliations:** 1 Instituto de Investigación, Desarrollo e Innovación en Biotecnología Sanitaria de Elche (IDiBE), Universidad Miguel Hernández de Elche, 03202 Alicante, Spain; pbadia@umh.es (P.V.B.-H.); m.fuentesb@umh.es (M.F.-B.); mlosada@umh.es (M.L.-E.); r.diaz@umh.es (R.D.-P.); a.mira@umh.es (A.M.); msaceda@umh.es (M.S.); pgarcia@umh.es (P.G.-M.); 2 Fundación para El Fomento de la Investigación Sanitaria y Biomédica de la Comunidad Valenciana (FISABIO), Hospital General Universitario de Elche, Unidad de Investigación, 03203 Alicante, Spain

**Keywords:** nanofibers, PMVEMA, electrospinning, doxorubicin, temozolomide, carmustine, glioblastoma

## Abstract

**Background/Objectives**: The local release of antineoplastic drugs in post-surgical treatments is an alternative way to improve their effectiveness against glioblastoma reappearance. Thus, it was proposed to develop a local delivery system based on electrospun PMVEMA-derived nanofibers for the administration of carmustine (BCNU), temozolomide (TMZ), and doxorubicin (DOX). **Methods**: Electrospun nanofibers were prepared using PMVEMA-monoethyl ester (PMVEMA-Es) and PMVEMA-acid (PMVEMA-Ac), loading BCNU, TMZ, and DOX at 1% or 8% (*w*/*w*). Their morphology, encapsulation efficiency, and release profiles were characterized by FESEM, confocal microscopy, and HPLC. Their biological effects were evaluated through cell viability, cell cycle, and intracellular accumulation assays in established cell lines from glioblastoma patients (HGUE-GB) and human astrocytes (HAs). **Results**: The nanofibers were optimized without defects, and encapsulation efficiencies were above 80%. The release studies showed a rapid initial release in the first hour, being DOX > TMZ > BCNU, while the second release rate was sustained in the cases of PMVEMA-Ac/TMZ (0.14%/h) and PMVEMA-Es/BCNU (1.2%/h), highlighting that, after 24 h under physiological conditions, the degradation of the loaded drug was lower than its free state, comparable to the Gliadel release system. Furthermore, it was confirmed that there was a dose-dependent decrease in cell viability for PMVEMA-Es/BCNU and PMVEMA-Ac/DOX, with higher cytotoxicity than free DOX. Finally, the lowest concentration tested had a relatively low effect on HAs compared with its effect on glioblastoma cells. **Conclusion**: PMVEMA-based electrospun nanofibers are effective in encapsulating and releasing antineoplastic drugs, suggesting their potential as a local delivery system to improve glioblastoma post-surgical treatment efficacy.

## 1. Introduction

Malignant tumors related to the brain and nervous system occupy the 17th position in terms of incidence, according to the Spanish Network of Cancer Registries, with more than 4000 new cases diagnosed annually [1]. Seventy-seven percent of these tumors are gliomas, among which astrocytomas are the most common type. The incidence of these tumors has increased in the last three decades, with glioblastomas being the most prevalent in adults, representing 67% of all gliomas diagnosed and only 7% in children [2].

The one-year survival is only 37.2% following current therapies, such as Stupp, which combine radiotherapy and temozolomide (TMZ) administration post-surgery, and 5% at five years. This may be due to conventional methods of drug administration, which have limitations related to repeated dosing and high systemic toxicity, for example, the cardiotoxicity of doxorubicin (DOX) used in neuroblastoma treatments. For this reason, new drug delivery systems are being developed, optimizing the therapeutic benefits of the drugs [3,4].

In this context, the development of new drug delivery systems based on soft biomaterials is emerging as a promising solution to optimize oncological treatments. These systems allow a controlled and localized release of drugs, minimizing systemic adverse effects and maximizing therapeutic efficacy. An example of this advance is Gliadel^®^ polymeric wafers, manufactured by Eisai. The delivery system is based on a biodegradable sebacic acid-based polyconjugate implanted directly into the tumor cavity after surgery, which releases carmustine (BCNU) in a sustained manner to treat glioblastoma [5,6].

However, the need for innovation in the form of drug delivery as a treatment for glioblastoma is imminent. In recent decades, the use of these types of polymeric biomaterials as delivery systems has been increasing. Likewise, nanostructured soft biomaterials, such as polymeric electrospun nanofibers, have stood out for their high encapsulation efficiency, high malleability, and low processing and storage costs. These materials have a relative porosity, behaving as synthetic extracellular matrices, allowing the passage of ions and intercellular communication molecules. Thanks to these properties and their dual ability to have fast or slow degradation, they can be applied locally and overcome the limitations of the blood–brain barrier (BBB) involved in systemic drug delivery [7,8].

The use of biocompatible and biodegradable polymers in nanofiber synthesis can provide malleable matrices with low or no toxicity to the body. Polymers such as poly (methyl vinyl ether-alt-maleic anhydride) or PMVEMA, and its derivatives such as PMVEMA-monoethyl ester (PMVEMA-Es) and PMVEMA-acid (PMVEMA-Ac), have been approved by the U.S. Food and Drug Administration (FDA) and characterized for application as copolymers in biomedical applications and as drug delivery systems [9,10].

In previous works, PMEVEMA has been used to develop nanofibers by electrospinning to encapsulate active compounds with biomedical applications, such as antibiotics, capsaicin, methyl salicylate, or salicylic acid, among others [10,11]. Likewise, PMVEMA and other polymers such as poly(lactic-co-glycolic acid) (PLGA) have been used to encapsulate drugs with fluorescent properties, such as 5-aminolevulinic acid (5-ALA) and DOX. This allows treatment monitoring before and during surgery [12,13].

To address these challenges, this work proposes the development of a local delivery system based on electrospun PMVEMA nanofibers for the administration of antineoplastic drugs. TMZ and BCNU were selected due to their current application in glioblastoma therapy, with the Gliadel wafer being a reference in post-surgical release systems applied to the central nervous system [14]. Furthermore, to track the encapsulated drugs, DOX was chosen due to its fluorescent properties. Since this drug cannot cross the blood–brain barrier (BBB) and has a high systemic toxicity, the local administration of the drug could overcome its limitations in clinics [15,16]. Such a delivery strategy has the potential to increase drug bioavailability, allow both rapid and sustained controlled release, and reduce side effects associated with cancer treatments. Analyses of the morphological characteristics, encapsulation efficiency, and drug release profiles, and a range of cellular assays were conducted for this purpose.

## 2. Materials and Methods

### 2.1. Reagents

PMVEMA-Ac polymer, with a weight average molecular mass (Mw) of 1,980,000 g/mol, and PMVEMA-Es, with a molecular mass by weight of 130,000 g/mol (Sigma-Aldrich, St. Louis, MO, USA), were used for the solutions used in the preparation of nanofibers. The drugs TMZ, BCNU, and DOX were purchased from TCI Europe N.V. (Antwerp, Belgium). Gliadel^®^ wafers were acquired from Eisai Co., Ltd. (Tokyo, Japan). The solvents used were acetonitrile (ACN-Sigma-Aldrich, St. Louis, MO, USA) and methanol (MeOH-Merck KGaA, Darmstadt, Germany), both high-performance liquid chromatography (HPLC)-grade and, in their case, mass-grade (LC/MS), as well as distilled and deionized water with a Milli-Q apparatus (Millipore, Darmstadt, Germany).

### 2.2. Preparation of Drug-Loaded PMVEMA-Ac or PMVEMA-Es Nanofibers

The optimum PMVEMA concentrations for electrospinning were determined in previous studies by our group, being 20% (*w/w*) in an aqueous solution for Ac and 25% (*w*/*w*) for Es in ethanol [13]. The solution was left under constant stirring at room temperature overnight. Once homogeneous, TMZ, DOX, or BCNU were added to the PMVEMA-Ac solution at 1% (*w*/*w*) with respect to the polymer. Meanwhile, to improve the encapsulation ratio of BCNU to higher than that using Gliadel^®^ (3.85% *w*/*w*) [14], PMVEMA-Es was used due to its solubility in ethanol. This allowed the encapsulation of the drug up to 8:25% *w*/*w* with respect to the polymer. Once the solution was homogeneous, it was loaded into a BD Discardit II two-piece 2 mL plastic syringe with a 316 stainless-steel, 18-gauge, blunt-tipped needle with a 90° angle (Sigma-Aldrich, St. Louis, USA) and placed on a KDScientific-100-CE infusion pump (mL/h) (KD Scientific, Holliston, MA, USA). An electrostatic field was then generated between the needle and the collector (cm) by applying a specified voltage (kV) using a Glassman High Voltage FC60P2 high-voltage source (Glassman High Voltage Inc., Salem, NJ, USA). The optimal electrospinning parameters for the loaded PMVEMA-Ac nanofibers were 16 kV, 10 cm, and 0.25 mL/h, and for the loaded PMVEMA-Es nanofibers, the parameters were set at 6.8 kV, 10 cm, and 0.25 mL/h. The environmental conditions in which this procedure was performed were at room temperature and a relative humidity of 40–50%.

### 2.3. Loaded Electrospun Nanofibers’ Characterization

#### 2.3.1. Field-Emission Scanning Electron Microscopy (FESEM)

For morphological analysis of the nanofibers, photographs were taken using a SIGMA 300 VP model Schottky hot cathode field-emission microscope (ZEISS) (Oberkochen, Germany). The photographs obtained by FESEM were analyzed with the ImageJ program (version 1.54d), measuring the diameter of 100 nanofibers to obtain an average of their sizes, with the required scale adjustment.

#### 2.3.2. Confocal Microscopy

The fluorescent properties of DOX allowed the characterization of the composition of PMVMEA-Ac/DOX 1% nanofibers by fluorescence microscopy. For this purpose, an LSM900 confocal microscope with Airyscan 2 ZEISS (Oberkochen, Germany) was used. The fluorescence intensity was recorded at the emission wavelength of 590 nm, with an excitation wavelength of 480 nm.

#### 2.3.3. High-Performance Liquid Chromatography

Chromatographic analyses were conducted using a Nexera UHPLC system (Shimadzu Scientific Instruments, Inc., Tokyo, Japan) consisting of a DGU-405 degasser, an LC-40D × 3 binary pump, an SIL-40C autosampler, a CTO-40C column oven, an SPD-30A detector, and a CBM-40 system controller. Chromatographic separation was performed with a LiChrospher^®^ RP-18 (4 × 250 mm; 5 μM) for TMZ analysis. Meanwhile, for DOX and BCNU separation, a Poroshell 120 SB-C18 column (4.6 × 150 mm; 2.7 μM) (Agilent Technologies, Inc., Palo Alto, CA, USA) was used. The characteristics of the methods used for each piece of equipment are described in Table 1.

Standard curves were generated for each antineoplastic agent using a range of concentrations for each one (Figure S1), and the areas under the curves of the chromatogram peaks for each sample were integrated and plotted on the corresponding calibration curve to determine the total concentration (Figure S2).

#### 2.3.4. Encapsulation Efficiency Study and Drug Loading

For this assay, the loaded PMVMEA-Ac and PMVEMA-Es nanofibers were dissolved in water and ethanol, respectively. The encapsulation efficiency (EE%) in the nanofiber drug was calculated against the theoretical maximum estimate. For quantification, a curve of free drug concentrations was generated, and the values obtained were interpolated on the line. To inject the samples into the HPLC and determine the amount of loaded compound, the nanofiber solutions were filtered through a 0.45 µm BRANCHIA nylon syringe filter (Barcelona, Spain).

Equation (1). Equation to calculate encapsulation efficiency.

(1) $$\mathrm{EE}\%=\frac{\mathrm{Real}\;\mathrm{concentration}}{\mathrm{Theorethical}\;\mathrm{concentration}}\times \;100\%$$

Equation (2). Equation to calculate drug-loading capacity.

(2) $$\mathrm{DL}\%=\frac{\mathrm{Mass}\;\mathrm{of}\;\mathrm{drug}\;\mathrm{encapsulated}}{\mathrm{Total}\;\mathrm{mass}\;\mathrm{of}\;\mathrm{nanofibers}}\times \;100\%$$

#### 2.3.5. Drug Release Assay

The assay was performed with a final volume of 2 mL of 0.1× DPBS (Dulbecco’s phosphate-buffered saline), in a space of 5 cm^3^, at pH 7.4 and at 37 °C. An amount of 100 μL was recovered, maintaining the initial volume. Samples were recovered at specific times, at 0.25, 0.5, 0.75, 1, 2, 4, and 24 h, in triplicate. The final concentrations were calculated by Equation (3), which considers the volume extracted at different times.

Equation (3). Equation to calculate the amount of drug released over time.

(3) $${\mathrm{Cn}}_{\mathrm{total}}=\frac{C_{n}\;\times \;V_{T}+\sum_{i=1}^{n-1}{(C}_{i}\;\times \;V_{s})}{V_{T}}$$

In the equation, *Cn_total_* = the cumulative concentration of the drug released at time *n*; *C_n_* = the measured concentration of the drug released at time *n*; *V_T_* = the total volume of the release medium; C_i_ = the concentration measured in the sample taken at the previous time; and V_S_ = the volume extracted at each point. The concentrations obtained were corrected considering the degradation of the drug at the established time, recovering and analyzing free drug samples under the same experimental conditions. Subsequently, the average release rate of the samples was determined using the following equation:

Equation (4). Equation to calculate the average release rate.

(4) $${\mathrm{Release}}_{\mathrm{rate}\;\left(t_{1}-t_{2}\right)}=\frac{C_{t2}-{\;C}_{t1}}{t_{2\;}-{\;t}_{1}}$$

### 2.4. Cellular Assays

#### 2.4.1. Cell Cultures

For the in vitro study of the antineoplastic drug properties of PMVEMA-Ac/Es nanofibers loaded with DOX, BCNU, or TMZ, the HGUE-GB glioblastoma multiform cell lines established from primary cultures of glioblastoma patients from the Hospital General Universitario de Elche were used. The lines HGUE-GB-16, 18, 37, 39, 40, 42, and 48 were characterized based on their sensitivity to radiotherapy, TMZ, and BCNU by the Chemoresistance and Cancer Group of the Institute for Research, Development, and Innovation in Health Biotechnology of Elche (IDiBE) [17].

Glioblastoma cell lines were cultured with DMEM-F12 medium (Dulbecco’s modified Eagle’s medium; Nutrient Mixture F12) rich in glucose, with 2 mM stable glutamine, 25 mM Hepes (Biowest) (Riverside, CA, USA), 10% fetal bovine serum from Biowest (FBS), and 1% antibiotic (penicillin and streptomycin). The culture conditions were 37 °C with 5% CO_2_ and a humidified atmosphere in an incubator (ESCO Lifesciences GmbH, Friedberg, Germany).

Human adult astrocytes (HAs) (Cat. No. 882A-05a) were obtained from Cell Applications, Inc. (San Diego, CA, USA) and cultured according to the manufacturer’s instructions. Cells were maintained in HA Growth Medium (Cat. No. 821-500, Cell Applications, Inc., San Diego CA, USA) under standard conditions (37 °C; 5% CO_2_). Subculturing was performed every 2 to 3 days using 0.25% trypsin. For 96-well plate assays, cells were seeded at a density of 20,000 cells per well.

#### 2.4.2. MTT Assay

The effects on cell viability of PMVEMA-Ac/Es nanofibers with DOX, TMZ, or BCNU, considering 100% of EE, were analyzed with the MTT (3-(4,5-dimethylthiazol-2-yl)-2,5-diphenyltetrazolium bromide) colorimetric assay. Cells were seeded in 96-well plates at a density of 4000 cells per well. After 24 h, the treatments were added in sextuplicate. After 72 h of incubation with the appropriate treatments, MTT reagent (Sigma Aldrich^®^, St. Louis, MO, USA) was added at a final concentration of 0.25 mg/mL for a period of 3 h. The content of the wells was then replaced with 200 μL of dimethyl sulfoxide (DMSO, Sigma Aldrich^®^, St. Louis, MO, USA). Finally, the absorbance was measured at 570 nm with an Eon™ microplate reader (BioTeK^®^, Winooski, VT, USA). Polymeric nanofiber controls (drug-free) were added at equivalent weights to those used in the drug-loaded treatments.

#### 2.4.3. Cell Cycle

Flow cytometry analysis was used to determine the effect on the cell cycle. Initially, cells were seeded in a 6-well plate at a density of 300,000 cells per well. After 24 h, the treatment was applied and maintained for an additional 24 h. Cells were then trypsinized and centrifuged at 400× *g* for 5 min, and then the cell pellet was resuspended in 1 mL of 75% ethanol.

Ethanol-fixed cells were maintained at −20 °C until cell cycle analysis. Cells were first centrifuged at 400× *g* for 5 min to remove ethanol and resuspended in PBS buffer with 0.5% (*v*/*v*) Triton X-100 (Sigma Aldrich^®^) to permeabilize the plasma membrane, 25 μg/mL RNase A (Sigma Aldrich) to remove RNA, and 25 μg/mL propidium iodide (Promocell, Heidelberg, Germany) to label DNA. The cells were incubated in the dark at room temperature for 30 min, and subsequently, cell cycle analysis was performed using the BD FACSCanto™ II flow cytometer (BD Biosciences, San Jose, CA, USA).

#### 2.4.4. Intracellular Accumulation Assay

Cells were seeded in 96-well plates at a density of 9000 cells per well. After 24 h, free and encapsulated DOX were added to them at different concentrations and incubated for 10, 20, 30, and 60 min to determine the minimum time for DOX saturation within the cells. The cells were then treated with Hoechst 33,342 (lambda excitation wavelength: 352 nm and lambda emission wavelength: 454 nm) (Thermofisher, Waltham, MA, USA) at 2000 ng/mL for 20 min. Subsequently, the cells were washed with phosphate-buffered saline (PBS). The fluorescence intensity of the compounds was measured using a Cytation multimode microplate reader (BioTek, Winooski, VT, USA).

### 2.5. Statistical Analysis

The data obtained were analyzed using two-way ANOVA statistical tests, nonlinear regression, or a Gaussian function, as appropriate. The GraphPad Prism 8.0 software (La Jolla, CA, USA) was used for this and the graphing of the results. The results are represented as means and standard deviations. Statistically significant differences between experimental groups are shown as * (*p* < 0.05), ** (*p* < 0.01), *** (*p* < 0.001), and **** (*p* < 0.0001).

## 3. Results

### 3.1. Morphological Characterization of Loaded Nanofibers

**Figure 1:**
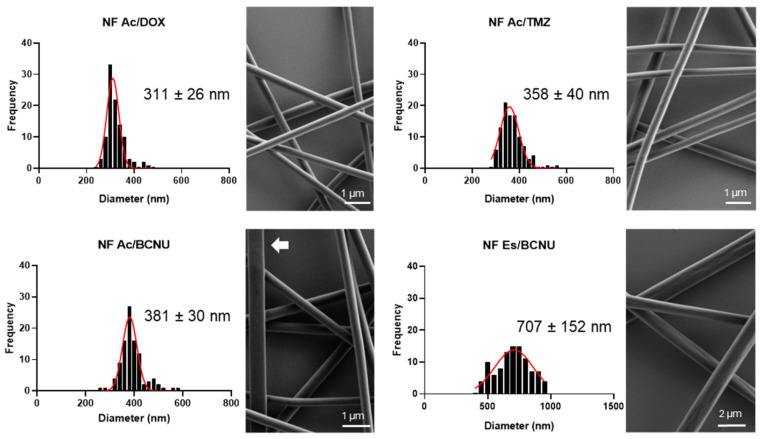
FESEM analysis of electrospun PMVEMA-Ac nanofibers with 1% DOX, TMZ, or BCNU and PMVEMA-Es with 8% BCNU (*w*/*w*). (**Left**) Frequency histograms of nanofiber diameters (*n* = 100; mean ± SD). (**Right**) FESEM photographs; the presence of ribbons is highlighted with a white arrow.

Initially, the diameters of the nanofibers were characterized with a ratio of 20:1% drug, *w*/*w* against the PMVEMA-Ac. We obtained diameters of 311 ± 26, 356 ± 40, and 381 ± 30 nm with DOX, TMZ, and BCNU, respectively. The samples did not show deformities or visible defects beyond the formation of ribbons with BCNU at 1% (Figure 1).

To improve the encapsulation ratio of BCNU, we used PMVEMA-Es due to its solubility in ethanol. This allowed us to encapsulate the drug up to 8:25% *w*/*w* with respect to the polymer. The electrospinning parameters were set at 6.8 kV, 10 cm, and 0.25 mL/h. PMVEMA-Es nanofibers loaded with 8% BCNU showed an average diameter of 707 ± 152 nm (Figure 1). This measure is related to the polymer without the drug.

**Figure 2:**
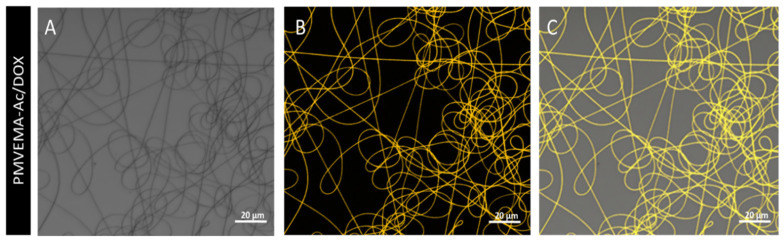
Photographs taken by confocal microscopy of PMVEMA-Ac/DOX 1% nanofibers. (**A**) Brightfield, (**B**) fluorescence, and (**C**) overlay of A and B. λexc = 475 nm; λem = 590 nm.

The qualitative assay of DOX loaded in the nanofibers was made with confocal microscopy due to the fluorescent properties of DOX. The nanofibers were excited at the wavelength of 470 nm. The analysis of the PMVEMA-Ac nanofibers showed no fluorescence; however, when DOX was encapsulated in the polymeric nanofibers, fluorescence was observed at its emission length of 590 nm (Figure 2), thus confirming the homogeneous distribution of DOX within the nanofibers.

### 3.2. HPLC Quantification of Encapsulation Efficiency and Drug Release Assay from Loaded Nanofibers

Initially, the incorporation of the drugs into the nanofibers was studied by FTIR, in some cases observing the presence of distinct drug peaks within the spectrum (Figures S3 and S4). Consequently, the encapsulation efficiency was determined by HPLC, determining a value of 100% for TMZ and DOX in PMVE-MA-Ac and values of 80 and 81% for BCNU in PMVEMA-Ac and PMVEMA-Es nanofibers, respectively (Table 2). Since the PMVEMA-Es/BCNU nanofibers had an 80% EE and a 6.5% DL, which was higher than the Gliadel wafers’ encapsulation ratio [14], the material was further characterized, discarding the PMVEMA-Ac/BCNU 1% nanofibers’ characterization.

**Figure 3:**
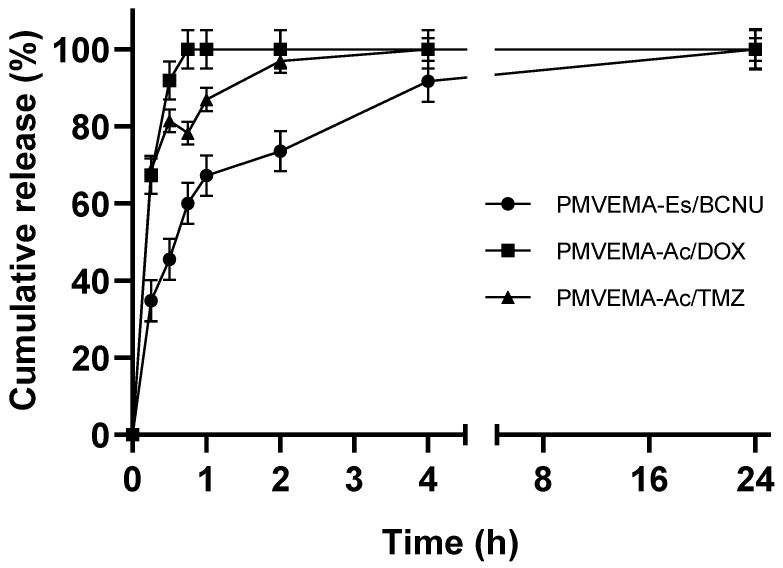
Cumulative release of the drugs, corrected with respect to drug degradation in the free state. The drugs BCNU in PMVEMA-Es nanofibers and TMZ or DOX in PMVEMA-Ac nanofibers are shown with a release at 37° and pH 7.4 during 24 h. Data are shown as means ± CVs (*n* = 3; mean ± SD).

Considering the encapsulation efficiency of the nanofibers, a release test was performed for these systems. In the test, the polymeric nanofibers showed a double release. The first one was very fast until 1 h, and the second one slowed down from 2 to 24 h. The PMVEMA-Es/BCNU nanofibers showed a slower release during the first hour compared with PMVEMA-Ac/DOX or TMZ nanofibers (Figure 3). In the case of BCNU, the release kinetics were confirmed with the Bratton–Marshall colorimetric assay, observing the same release trend (Figure S5). When analyzing the fit of the data to kinetic models, it was observed that the release of both PMVMEA-Ac and PMVEMA-Es fit a first-order model and a Korsmeyer–Peppas model, with the first-order model being the best fit for PMVEMA-Ac loaded with 1% DOX (Table S1).

When we analyzed the average release rates of the samples, we observed that 67.2%/h of BCNU was released from the PMVEMA-Es nanofibers in the first hour, and, subsequently, between 2 and 24 h, the ratio decreased to 1.2%/h. This dual effect was also observed in the release of TMZ, with ratios of 86.9%/h and 0.14%/h, with a faster initial release. However, the ratio for DOX was 100%/h in the first hour.

**Figure 4:**
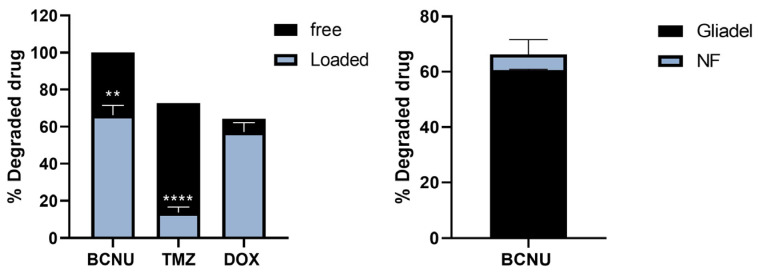
Degradation of encapsulated and free drug at 24 h. (**left**) The percentages of degraded drug for BCNU, TMZ and DOX. (**right**) The percentage of BCNU degradation encapsulated in PMVEMA-Es nanofibers and Gliadel^®^ is shown. The assay conditions were 37°, at 24 h and pH 7.4. (*n* = 3; mean ± SD; ** *p* < 0.01; **** *p* < 0.0001).

After one day under physiological conditions, the degradation of BCNU decreased by 33.7% when it was encapsulated in the nanofibers, while in free format, it was totally degraded. A similar effect occurred with encapsulated TMZ and DOX, observing decreases in drug degradation of 59% and 7.1%, respectively, compared with the free state. In the case of BCNU, the release from PMVEMA-Es nanofibers was compared against the release from the Gliadel^®^ wafer, observing a 5.66% difference in drug degradation, without this difference being statistically significant (Figure 4).

### 3.3. Evaluation of Biological Effect of Drug Loading

#### 3.3.1. Cell Viability Evaluation by MTT Test in HGUE-GB Cell Lines

**Figure 5:**
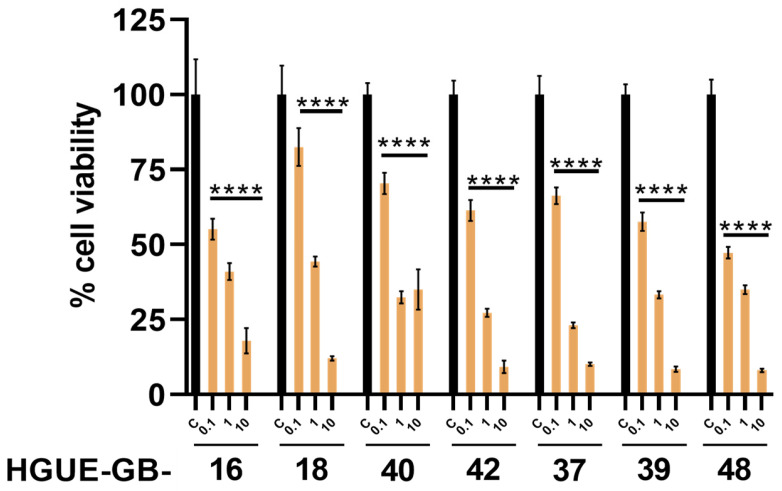
Cell viability by MTT of HGUE-GB treated with different DOX concentrations (μM). In all cases, the decrease in cell viability was statistically significant with respect to the control (**** *p* < 0.0001). Percent cell viability is shown relative to untreated control (C) (*n* = 6; mean ± SD).

Initially, the effect of DOX on the viability of the cell lines HGUE-GB-16, 18, 37, 39, 40, 42, and 48 was evaluated. The results show a statistically significant decrease at 0.1 μM in the cell viability of all the cell lines, highlighting HGUE-GB-48 as the line that, at 0.1 μM, reduced cell viability to less than 50%. Meanwhile, the HGUE-GB-18 line stood out as the least sensitive, since only 20% of its cell viability was reduced with the lowest concentration applied, being 0.1 μM (Figure 5). Since all cell lines tested showed sensitivity to DOX, HGUE-GB-37 and HGUE-GB-42 were selected for biological assays based on previous reports describing their sensitivity and partial sensitivity, respectively, to BCNU treatment [18].

**Figure 6:**
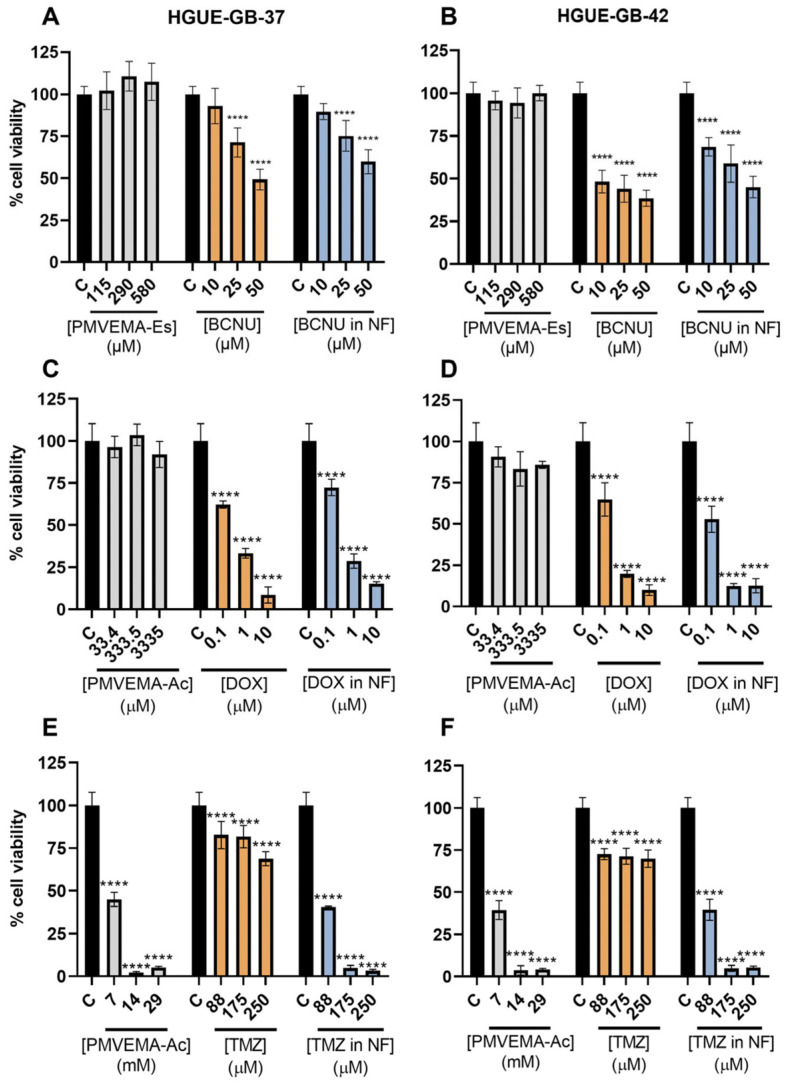
Viability of HGUE-GB-37 (**A**,**C**,**E**) and HGUE-GB-42 (**B**,**D**,**F**) cells treated with different concentrations of nanofibers without drug (grey), free drug (orange), and encapsulated drug (blue) at 72 h post-treatment. Polymer concentration (µM or mM) reflects the amount employed in treatments with the encapsulated drug. Cell viability is shown as a percentage relative to the untreated control (**** *p* < 0.0001) (*n* = 6; mean ± SD).

Once the cell lines were selected, the effect of the encapsulated BCNU was evaluated. No significant differences were observed between free BCNU and encapsulated BCNU in HGUE-GB-37. However, in HGUE-GB-42, a slight decrease in cell viability was observed at the highest concentration of encapsulated BCNU compared with free BCNU (Figure 6A,B). Similarly, after DOX treatment, a dose-dependent decrease in cell viability was observed for both the free and encapsulated forms. In this case, the minimum concentration required to produce a significant effect was 0.1 µM in both cell lines, with 10 µM being the highest concentration tested. No significant differences were observed between the free and encapsulated forms (Figure 6C,D). In the BCNU and DOX treatments, the polymer PMVEMA-Es or PMVEMA-Ac, alone, showed no significant effect on cell viability.

In the case of TMZ, it was observed that the polymer at a concentration of 7 mM reduced the viability of the cell lines by more than 50%. Meanwhile, with 250 µM TMZ, a significant decrease in cell viability was observed; however, it did not reach a 50% decrease. As for the effect of encapsulated TMZ, a significant decrease in cell viability was observed, which can be attributed to the effect of the polymer (Figure 6F,G). Based on the results obtained, the encapsulated TMZ was excluded from the following characterization.

#### 3.3.2. Evaluation of Effect of Drug Loading on HGUE-GB Cell Cycle

**Figure 7:**
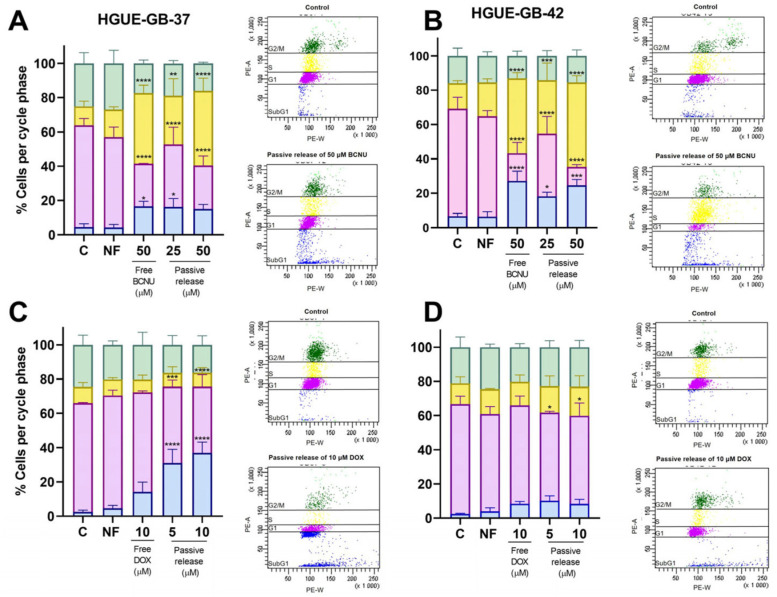
Effect of the passive release of encapsulated BCNU and DOX on the cell cycle of HGUE-GB-37 and HGUE-GB-42 cell lines 24 h post-treatment. Control cells, with drug-free nanofibers, the free drug, and passive release of the drug from nanofibers, at equivalent concentrations, are shown. (**A**,**B**) Treated with BCNU at 25 and 50 µM. (**C**,**D**) Treated with 5 and 10 µM of DOX. Phases are represented as G2/M in green, S in yellow, G1 in pink, and SubG1 in blue (* *p* < 0.05, ** *p* < 0.01, *** *p* < 0.001, and **** *p* < 0.0001; *n* = 3; mean ± SD).

Once the effect of BCNU and DOX on the cell lines was established, we analyzed the effect of the encapsulated drugs on the distribution of the different phases of the cell cycle. For this purpose, free BCNU and PMVEMA-Es/BCNU 8%, and PMVEMA-Ac/DOX 1% nanofibers were applied directly to the culture medium at equivalent concentrations for a passive release effect. In addition, a control was included using PMVEMA nanofibers without the drug (Figure 7).

In the case of BCNU, the free and passive release drug induced a notable accumulation in the S phase in both cell lines, consistent with a cytostatic effect. In the HGUE-GB-37 cell line, the S phase increased by approximately 30% at 50 µM, respectively, for all BCNU treatments (Figure 7A). In HGUE-GB-42, the increase in the S phase was also evident, and no statistically significant differences between treatments were observed (Figure 7B). Overall, BCNU exhibited a primarily cytostatic effect through S-phase arrest 24 h post-treatment, with a modest cytotoxic component in HGUE-GB-42 at higher concentrations. The encapsulated form retained comparable activity to free BCNU.

Meanwhile, analysis of the effects of passively released DOX on the cell cycle of HGUE-GB-37 and HGUE-GB-42 cell lines revealed a statistically significant increase in the SubG1 population, indicative of cell death, compared with the control. In the HGUE-GB-37 cell line, the proportion of cells in the SubG1 phase was higher than that observed in both the control and the HGUE-GB-42 line. Notably, treatment with 10 μM of passively released DOX resulted in a 36.4% increase in the SubG1 population compared with the same concentration of free DOX. In contrast, no significant differences between treatments were observed in the HGUE-GB-42 line (Figure 7C,D).

#### 3.3.3. Cell Viability Evaluation by MTT Test in HA Cells

**Figure 8:**
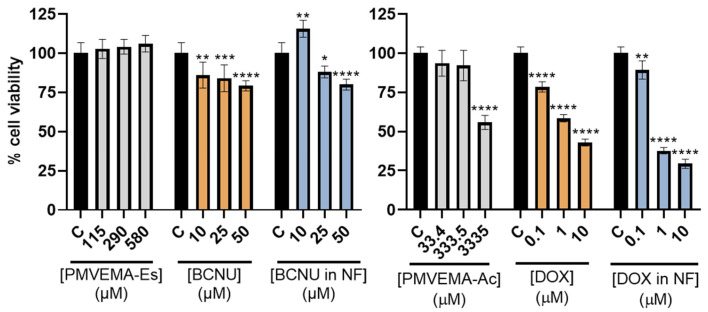
Viability of HA cell line treated with different concentrations of nanofibers without drug (grey), free drug (orange), and encapsulated drug (blue) at 72 h post-treatment. Cell viability is shown as percentage relative to untreated control (* *p* < 0.05; ** *p* < 0.01; *** *p* < 0.001; and **** *p* < 0.0001) (*n* = 6; mean ± SD).

Once the effect of the treatments on the glioblastoma lines was established, the effect on human astrocytes was evaluated to determine the therapeutic window of the treatment. In the case of PMVEMA-Es/BCNU, we observed that the polymer did not diminish astrocyte cell viability, even showing a statistically significant increase in cell viability. In the case of BCNU, cell viability decreased by up to 20% with respect to the control (Figure 8).

As for the treatment with PMVEMA-Ac/DOX, the polymer at high concentrations significantly decreased cell viability, without showing a significant effect at concentrations lower than 333.5 μM. Meanwhile, we found that DOX dose-dependently decreased cell viability up to 20%, in both free and encapsulated forms (Figure 8).

#### 3.3.4. Accumulation Test of Intercellularly Loaded DOX

**Figure 9:**
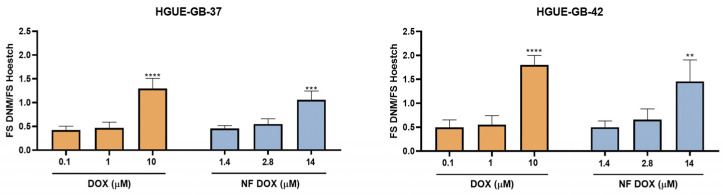
Intracellular accumulation in HGUE-GB-37 and GB-42 lines of encapsulated DOX. Cells were treated with different concentrations (μM) of free (black) and encapsulated (white) DOX for 20 min. The fluorescence intensity of DOX was normalized against Hoechst (*Y*-axis) (** *p* < 0.01; *** *p* < 0.001; and **** *p* < 0.0001) (*n* = 3; mean ± SD).

Finally, to continue with the evaluation of the effect of DOX on the cell lines, an accumulation assay was performed at 20 min for the concentrations studied to confirm that the encapsulated DOX retained its properties and internalized as it does in its free state. The intracellular accumulation was measured when the DOX fluorescence signal inside the cell reached saturation. The highest fluorescence intensities of encapsulated DOX and free DOX were between 14 and 10 μM, respectively. A fluorescence intensity difference of approximately 0.5 was observed between the two cell lines, with HGUE-GB-37 showing the lower signal (Figure 9).

## 4. Discussion

PMVEMA-derived polymeric nanofibers were proposed as a post-surgical local release system for BCNU, TMZ, and DOX. Initially, the nanofibers were synthesized, and their morphology was characterized (Figure 1), observing that the encapsulation of the drug did not change the electrospinning parameters of the matrix polymer, nor did they form visible defects in the nanofibers, which is consistent with the encapsulation of other compounds, such as 5-ALA [10,13,19]. Likewise, the diameter of the PMVEMA-Ac nanofibers remained between 300 and 400 nm. The narrowing and widening of the diameter may have been due to an alteration in the conductive properties of the solution due to the incorporation of the drug. It should be noted that the increase in the diameter of the polymeric nanofibers with BCNU can be associated with the hydrophobic properties of the drug [12,20]. However, the solubility compatibility of BCNU with PMVEMA-Es allowed us to increase its encapsulation percentage to 8%, being above the 3.85% loading efficiency, with respect to the polymer, of other clinical treatments, such as Gliadel^®^ [21]. Due to the fluorescent properties of DOX, the confocal microscopy technique was employed, observing DOX encapsulation homogeneously distributed in the nanofibers (Figure 2). This is a qualitative indicator that the nanofiber synthesis process does not affect the fluorescent properties of the drug, as has been reported for its encapsulation in other polymeric nanomaterials [12,22].

Initially, the FTIR spectrum of the loaded nanofibers was obtained, partially observing the presence of the drugs (Figures S3 and S4), which were confirmed by further assays. Meanwhile, the HPLC-UV technique was used to quantify the encapsulation efficiency in the nanofibers and the release assay. A 100% encapsulation efficiency was observed for DOX and TMZ in PMVEMA-Ac. Meanwhile, the values for PMVEMA-Ac/BCNU and PMVEMA-Es/BCNU nanofibers were 81 and 80%, respectively (Table 2). This 20% loss may be due to the low stability of the drug at room temperature during the electrospinning process. However, the encapsulation efficiency relative to the polymer is much higher than for other nanomaterials due to the nanofiber system [7,23,24]. A 24 h release assay was also performed, in which the polymeric nanofibers, both with PMVEMA-Ac and PMVEMA-Es, presented two different release rates, one from 0 h to 1 h and the other from 2 h to 24 h. In the first hour, the release rate was higher than 60%/h in all cases (Figure 3). The observed double release is consistent with the literature reporting on matrices formed with nanofibers, where the release rate could vary according to the number of nanofiber layers [25,26,27]. In thinner or shallower layers, a faster release was observed, while in deeper layers of the matrix, the release was slower due to the permeation of the aqueous medium. In the case of DOX, it only presented a fast release up to 1 h and may be subject to the same principle. In PMVEMA-Es nanofibers, which are more hydrophobic than PMEVEMA-Ac, the permeation of the medium is slower, which would delay drug release [28,29]. When analyzing the best fit of the classical models (Table S1), it was found that the PMVEMA-Ac/DOX 1% formulation followed first-order behavior and was, therefore, concentration-dependent. However, the fits for PMVEMA-Ac/TMZ 1% and PMVEMA-Es/BCNU 8% did not exhibit behavior that conformed to a specific model, aligning with first-order models and Korsmeyer–Peppas. Nevertheless, it appears that the dominant effect of very rapid release kinetics is driven by diffusion from the electrospun fiber mats due to their large surface area and high dissolution rates. In addition, randomly arranged fibers typically give rise to a rapid and burst release profile [30].

Similarly, the encapsulation of the drug and its biphasic release decreased the percentage of drug degradation over 24 h at pH 7.4 and 37° for all samples. The minor difference between the BCNU degradation (5.7%) released from the nanofibers against the Gliadel^®^ wafers (Figure 4) could indicate that this release system may have a similar clinical effect. That being said, DOX only decreased by 7.1%, whereas TMZ stood out with a 59% decrease compared with its free state (Figure 4), which could improve the efficacy of the treatment in the peri-tumor area [31,32].

As for the evaluation of the effect of DOX on the viability of HGUE-GB cell lines, we observed a significant decrease in cell viability at 0.1 μM at 72 h post-treatment, highlighting differences between the cell lines. This concentration was low when compared with those observed for the TMZ and BCNU treatments, establishing a high sensitivity of the lines to this drug (Figure 5). This effect can be related to multiple effects at the cellular level, generating reactive oxygen species, and interacting with DNA, interfering with the II-topoisomerase and the mitochondrial membrane [15,33]. Regarding the effect of encapsulated drugs compared with their free state in the selected cell lines HGUE-GB-37 and HGUE-GB-42, no significant differences were observed between the free or encapsulated drugs at 72 h post-treatments (Figure 6A–D). The DOX treatment resulted in a statistically significant dose-dependent decrease in cell viability at the free and encapsulated concentrations (Figure 6A–B). This result is comparable to other studies of drug encapsulation in polymeric nanostructures to treat glioblastoma [33,34]. In Figure 6C,D, it was observed that encapsulated BCNU had a greater effect on the HGUE-GB-42 line than on the HGUE-GB-37 line. This agrees with the cell sensitivity studies previously reported for these cell lines. Alternatively, at a concentration of 250 μM of TMZ, the reduction in cell viability was less than 50% in the cell lines. It would be necessary to use higher concentrations to achieve a significant cytotoxic effect [18]. However, this would represent a limitation at the 1:20 (drug–polymer) encapsulation ratio in PMVEMA-Ac, since a considerable decrease in cell viability was observed at a concentration of 7 mM. In this context, the effect observed after treatment with encapsulated TMZ could be due to polymer-induced acidification of the medium; therefore, it is not possible to differentiate between the effect induced by the drug and the effect induced by the polymer (Figure 6E,F). Although the nanofibers can stabilize TMZ in the medium up to 24 h (Figure 4), their low solubility in aqueous solutions at room temperature restricts the possibility of increasing the concentration of the encapsulated drug in the PMVEMA-Ac nanofibers [35].

The cell cycle study revealed that, at 24 h, passive release of BCNU showed a cytostatic effect, with a block in the S phase of the cell cycle, corresponding to a decrease in the percentage of cells in the G1 phase (Figure 7A,B). Meanwhile, the HGUE-GB-42 cell line had a higher cytotoxic effect compared with the HGUE-GB-37 line due to its reported sensitivity to this drug (Figure 7B) [17,36]. No differences were observed between treatments. Similarly, DOX was shown to have a cytotoxic effect at 24 h, with an increase in the SubG1 phase. In the HGUE-GB-37 line, it can be observed that the gradual release of DOX increased its cytotoxic effect linked to the increase in cells in the sub-G1 phase (Figure 7C), probably due to the release system. However, at 24 h in the HGUE-GB-42 line, no changes in cell populations per phase were observed (Figure 7D), which implies that the treatment window for this cell line could be between 24 and 72 h and is probably linked to antiproliferation effects. These differential results may be due to the intrinsic cell mechanisms of chemotherapy resistance [15,37,38,39].

In contrast, when the loaded nanofibers with HA cell lines were tested at 72 h post-treatment, free and encapsulated BCNU was observed to decrease cell viability by only 20% compared with an effect of more than 50% in glioblastoma lines (Figure 8). Similarly, PMVEMA-Es nanofibers did not decrease the viability of the astrocyte line, and there was even a statistically significant increase in cell viability at low concentrations. This effect could be linked to the intrinsic properties of PMVEMA-Es, which, like other biopolymers, can induce cell proliferation [40]. However, PMVEMA-Ac nanofibers loaded with DOX at 0.1 μM (the lowest concentration tested) decreased HA cell viability by 10% (Figure 8), compared with a decrease of more than 50% in glioblastoma lines. A difference of around 40% could be suitable for its application as a treatment [41,42].

Highlighting the effect of DOX on the cell lines, and thanks to its fluorescent properties, its characterization continued with the intracellular accumulation assay. In this test, it was observed that intracellular saturation was reached at 10 min, so that at 20 min, the highest fluorescence value was observed at 10 μM. A 0.5 difference in the fluorescence intensity was highlighted (Figure 9). This may be related to the possible chemoresistance of the HGUE-GB-37 line to DOX, linked to the expression of ATP-dependent membrane transporters (ABC) B1, B5, C2, and G2, reported for glioblastoma lines [37,38].

## 5. Conclusions

In the search for innovative treatments for glioblastoma, local delivery systems of antineoplastic drugs were approached as a promising strategy to reduce the recurrence rate in operated patients and the secondary effects of systemic administration. Electrospun PMVEMA-derivate nanofibers were able to encapsulate DOX and BCNU with a loading efficiency comparable to clinically approved versions of drug delivery systems. Under physiological conditions, PMVEMA nanofibers slowed down the degradation of free drugs, with initial fast release in the case of PMVEMA-Ac, while the rate decreased with PMVEMA-Es and a second sustained release. The biological results showed that both drugs maintained their antitumor properties after the encapsulation process. Cell viability was confirmed to be dose-dependent in two different primary glioblastoma cell lines, HGUE-GB37 and HGUE-GB-42. Furthermore, PMVEMA-Ac/TMZ nanofibers required cytotoxic polymer concentrations that did not allow their effect to be explored. It was established that the main effect of PMVEMA-Es/BCNU 8% nanofibers was cytostatic, while the effect of PMVEMA-Ac/DOX 1% nanofibers was cytotoxic. Their effect on the human astrocyte line showed a wide range of compatible applications for these nanofibers as delivery systems. It is noteworthy to point out that both the differences observed between the two tumor cell lines and astrocytes and the temporary treatments applied indicate the heterogeneity of this type of tumor. These results underline the potential of this delivery system, highlighting the importance of tailoring therapeutic strategies to the biological complexity of glioblastoma. Consequently, future work will focus on combining strategies based on these material platforms and, in particular, on developing more advanced 3D cell models to test the delivery systems and bring this research closer to clinical development.

## Figures and Tables

**Table 1:** Chromatographic methods of analysis for the three antineoplastic drugs.

Drug	Elution Mode	Organic Phase (%)	Detection (nm)	Column T° (°C)	Flow (mL/min)	Time (min)
BCNU ^1^	Gradient ^1^	45 to 50 MeOH	230	30	0.9	10
TMZ ^2^	Isocratic ^2^	30 MeOH	330	35	1.1	5
DOX	Gradient ^1^	20 to 70 ACN	480	40	1.0	5

^1^ = 0.1% formic acid; ^2^ = 0.5% acetic acid was added to the mobile phase.

**Table 2:** Percentages of encapsulation efficiency and drug loading of the nanofibers.

Polymer	Drug (%w/polymer)	EE (%)	DL (%)
PMVEMA-Ac	DOX 1	100	1.0
PMVEMA-Ac	TMZ 1	100	1.1
PMVEMA-Ac	BCNU 1	80	0.8
PMVEMA-Es	BCNU 8	81	6.5

## Data Availability

The data presented in this study are available upon request from the corresponding author.

## Supplementary Materials

The following supporting information can be downloaded at https://www.mdpi.com/article/10.3390/pharmaceutics17091172/s1. Figure S1. Calibration curves and equation used to calculate concentrations obtained by HPLC; Figure S2. Representative chromatograms obtained by HPLC-UV for the three studied drugs; Figure S3. FTIR spectra of PMVEMA-Ac, doxorubicin, and PMVEMA-Ac/Do × 1% nanofibers; Figure S4. Extended FTIR spectra from 2000 to 450 cm−1 corresponding to PMVEMA-ES, carmustine, or BCNU and PMVEMA-ES/BCNU 8% nanofibers; Figure S5. BCNU release comparison between free drug and that released from PMVEMA-Es 8% nanofibers at different times; Table S1. Best-fitting model of drug release kinetics analyzed for the loaded nanofibers.
