# Supplementary material for: Electrospun PMVEMA Nanofibers Developed as a Fast-Release Platform for Antineoplastic Drugs Tested in Glioblastoma Primary Cultures
Source: Pharmaceutics. 2025 Sep 8;17(9):1172. doi: 10.3390/pharmaceutics17091172 (PMC12473597; doi:10.3390/pharmaceutics17091172)
Supplement: Supplementary file 1 [file pharmaceutics-17-01172-s001.zip › pharmaceutics-3774303-supplementary.pdf]

pharmaceutics

MDPI

Supporting information

Electrospun PMVEMA Nanofibers Developed as a Fast-Release Platform for Antineoplastic Drugs Tested in Glioblastoma Primary Cultures

Pedro Valentín Badía-Hernández [1], Joan Moll Carrió [1], María Fuentes-Baile [1,2], María Losada-Echeberría [1], Rocío Díaz-Puertas [1], Amalia Mira [1], Miguel Saceda [1,2], Pilar García-Morales [1] and Ricardo Mallavia [1,*]

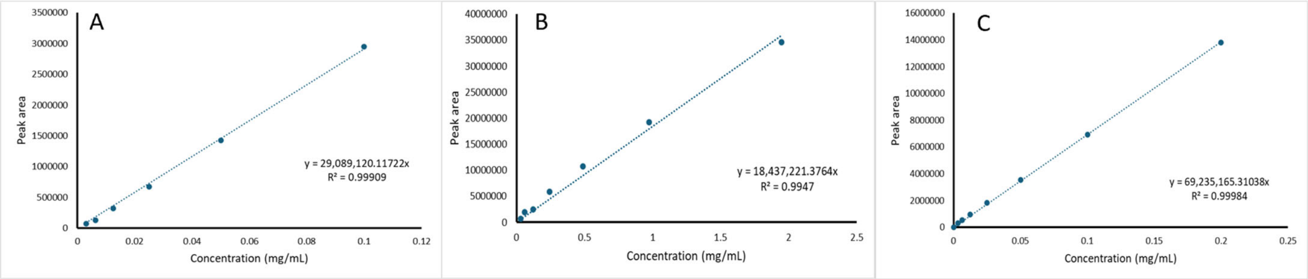

Figure S1. Calibration curves and equation used to calculated concentrations obtain by HPLC. A) BCNU calibration curve; B) DOX calibration curve; C) TMZ calibration curve.

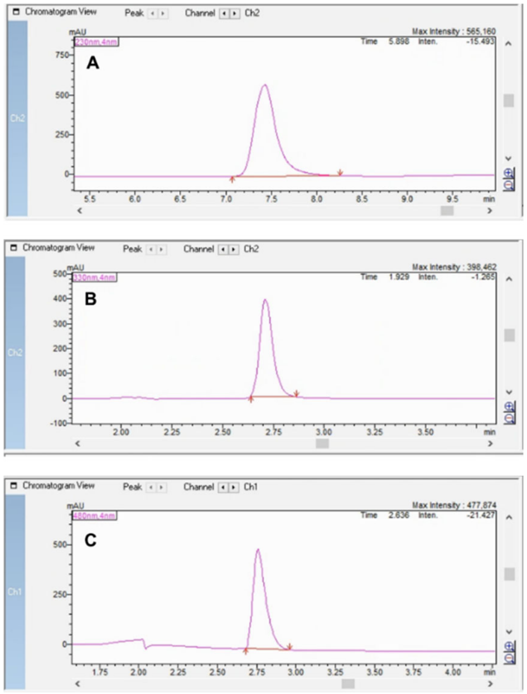

Figure S2. Representative chromatograms obtained by HPLC-UV for the three studied drugs. Integrated peaks corresponding to A) BCNU, B) TMZ, and C) DOX are shown. Each chromatogram indicates the retention time (minutes) and signal intensity expressed in mAU (milli-absorbance units), enabling the identification and quantification of each compound by ultraviolet (UV) detection.

Fourier transform infrared (FTIR) spectroscopy

The chemical composition of the samples was assessed through FTIR spectroscopy using a Spectrum Two™ FTIR spectrometer (PerkinElmer, Waltham, MA, USA). Two milligrams of the dried samples were pulverized with KBr salt at 25 ºC and compressed into a mold to create a pellet. The spectra (NS=32 scans) were captured within a frequencies range of 450–4000 cm^{-1} at a resolution of 4 cm^{-1}.

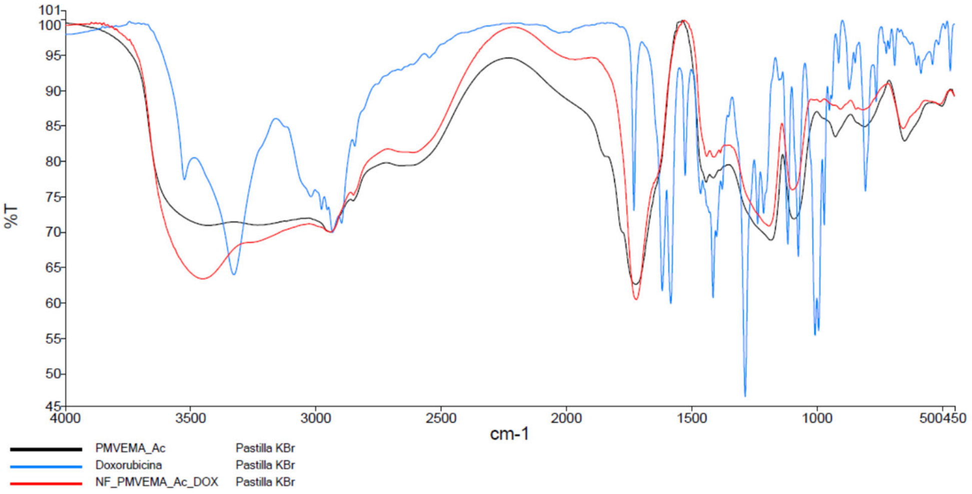

Figure S3. FTIR spectra of PMVEMA-Ac (black line), Doxorubicin (blue line) and PMEVMA-Ac/DOX 1 % (red line) nanofibers.

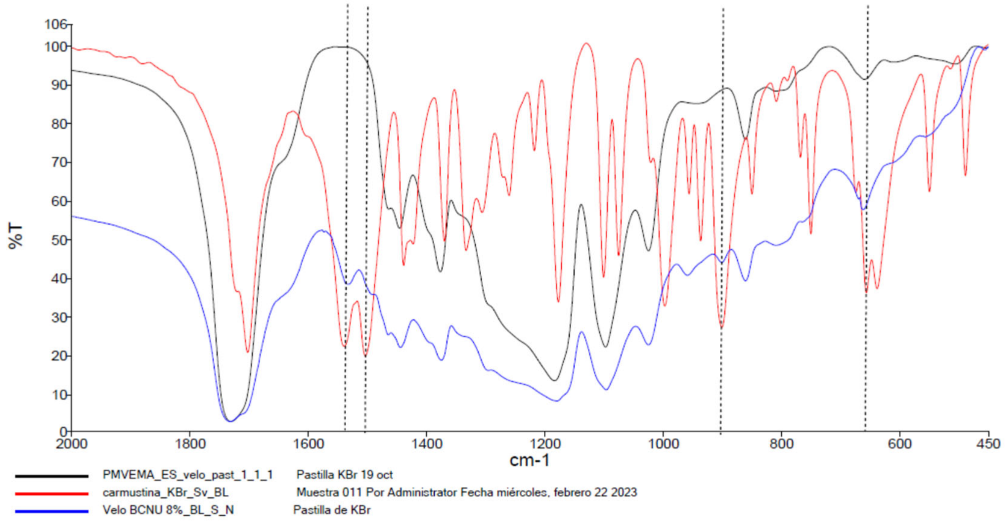

Figure S4. Extended FTIR spectra from 2000-450 cm^{-1}, corresponding to PMVEMA-Es (black line), carmustine or BCNU (red line), and PMEVMA-Es/BCNU 8 % nanofibers (blue line).

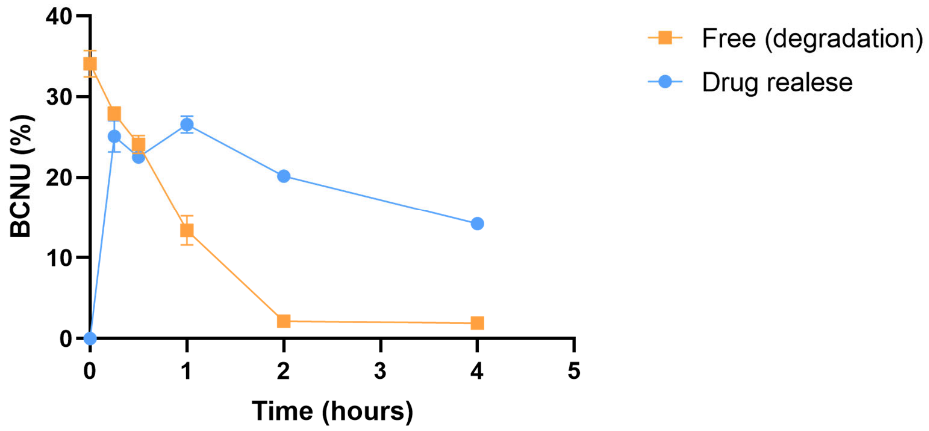

Figure S5. BCNU release comparison between free drug and released from PMVEMA-Es 8% nanofibers at different times. The percentage is shown relative to the theoretical BCNU added with a release at 37º and pH 7.4, during 4h. n=3 (mean ± SD).

Table S1. Best-fitting model of drug release kinetic analyzed for the loaded nanofibers.

Loaded nanofiber / R^2	**Zero-Order**	**First-order**	**Higuchi**	**Korsmeyer-Pepas**
PMVEMA-Es/BCNU8	0,8594	0,9465	0,8629	0,9463
PMVEMA-Ac/TMZ1	0,4282	0,9192	0,3743	0,9227
PMVEMA-Ac/DOX1	0,7917	0,9961	0,9836	0,9718
